# Incremental increases in physiological fluid shear progressively alter pathogenic phenotypes and gene expression in multidrug resistant *Salmonella*

**DOI:** 10.1080/19490976.2024.2357767

**Published:** 2024-05-23

**Authors:** Jiseon Yang, Jennifer Barrila, Eric A. Nauman, Seth D. Nydam, Shanshan Yang, Jin Park, Ami D. Gutierrez-Jensen, Christian L. Castro, C. Mark Ott, Kristina Buss, Jason Steel, Anne D. Zakrajsek, Mary M. Schuff, Cheryl A. Nickerson

**Affiliations:** a Biodesign Center for Fundamental and Applied Microbiomics, Arizona State University, Tempe, AZ, USA; b Biodesign Center for Immunotherapy, Vaccines and Virotherapy, Arizona State University, Tempe, AZ, USA; c School of Life Sciences, Arizona State University, Tempe, AZ, USA; d Department of Biomedical Engineering, University of Cincinnati, Cincinnati, OH, USA; e Department of Animal Care & Technologies, Arizona State University, Tempe, AZ, USA; f Bioinformatics Core Facility, Bioscience, Knowledge Enterprise, Arizona State University, Tempe, AZ, USA; g Biodesign Center for Personalized Diagnostics, Arizona State University, Tempe, AZ, USA; h JES Tech, Houston, TX, USA; i Biomedical Research and Environmental Sciences Division, NASA Johnson Space Center, Houston, TX, USA

**Keywords:** *Salmonella*, iNTS ST313/D23580, SPI-1/SPI-4, RNA-seq, RWV bioreactor, hydrodynamic biosystems/fluid shear

## Abstract

The ability of bacteria to sense and respond to mechanical forces has important implications for pathogens during infection, as they experience wide fluid shear fluctuations in the host. However, little is known about how mechanical forces encountered in the infected host drive microbial pathogenesis. Herein, we combined mathematical modeling with hydrodynamic bacterial culture to profile transcriptomic and pathogenesis-related phenotypes of multidrug resistant *S*. Typhimurium (ST313 D23580) under different fluid shear conditions relevant to its transition from the intestinal tract to the bloodstream. We report that D23580 exhibited incremental changes in transcriptomic profiles that correlated with its pathogenic phenotypes in response to these progressive increases in fluid shear. This is the first demonstration that incremental changes in fluid shear forces alter stress responses and gene expression in any ST313 strain and offers mechanistic insight into how forces encountered by bacteria during infection might impact their disease-causing ability in unexpected ways.

## Introduction

Multidrug resistant (MDR) bacteria are a global threat to public health. More than 2.8 million antimicrobial-resistant infections occur annually in the U.S. alone, resulting in over 35,000 deaths with risks increasing during the COVID-19 pandemic.^1,2^ The CDC categorized nontyphoidal *Salmonella* (NTS) as multidrug resistant pathogens that pose serious threats to human health.^1^ In the early 2000’s, highly invasive MDR *Salmonella enterica* serovars (iNTS) Sequence Type 313 (ST313) emerged in sub-Saharan Africa, causing life-threatening and often fatal bloodstream infections among immunocompromised populations.^3–9^ It has been reported that antimicrobial resistance and genome degradation contributed to ST313 epidemic outbreaks in Africa.^5^ ST313 variants are spreading globally and now found in Europe, Asia, and South America.^10–14^ While -omics profiling and phenotypic studies have examined a broad range of pathogenesis-related characteristics in ST313 lineages – including chromosomal and plasmid characterization,^5,15,16^ drug resistance,^5,12,17,18^ motility,^19,20^ biofilm formation,^21,22^ metabolism,^20,23^ virulence,^17,20,24,25^ *in vivo* tissue distribution,^20–26–30^ and host immune responses^31–34^ little is known regarding how fluid shear levels relevant to those encountered by these pathovars in the infected host regulate the infectious disease potential of this lineage.

*Salmonella* pathovars experience dynamic changes in fluid shear forces in their natural ecosystems, including in the infected host, ranging from low fluid shear in certain regions of the intestinal tract (*e.g.*, between the brush border microvilli^35,36^) to high fluid shear in the bloodstream.^35–38^ While it has become increasingly clear that fluid shear plays an important role in regulating a wide variety of pathogen characteristics that are important for the infectious disease process,^35–39–48^ most studies have not cultured bacteria under physiological fluid shear conditions that are routinely encountered during host-pathogen interactions. Indeed, the first demonstration that a physical force (fluid shear) could alter bacterial virulence was established using classic gastrointestinal *S*. Typhimurium strain χ3339, a mouse-passaged derivative of SL1344.^49,50^ This work, which used the dynamic Rotating Wall Vessel (RWV) bioreactor, established that fluid shear is a novel environmental regulator of bacterial virulence in mice, and that low fluid shear forces akin to those in the intestine increased the virulence, enhanced multiple pathogenesis-related stress responses, and altered transcriptomic and proteomic profiles of χ3339 in unexpected ways that are not observed during conventional culture conditions.^49^ During this previous study, the RWV was used in two different positions (hereafter referred to as the “*standard RWV configuration*”), with one bioreactor rotating perpendicular to the benchtop (resulting in a quiescent, low fluid shear optimized suspension culture), and the other bioreactor reoriented to rotate parallel to the benchtop resulting in the loss of low fluid shear suspension culture.

In subsequent work using χ3339, the RWV was modified by the inclusion of different sized beads to incrementally increase fluid shear levels ranging from lower (<0.01 dynes/cm^2^) to higher fluid shear levels (7.8 dynes/cm^2^).^51^ These fluid shear forces mimic those encountered by *Salmonella* in the infected host between the brush border microvilli in the intestine^36^ and the bloodstream, respectively.^20–52–56^ In response to these incrementally increasing fluid shear conditions, χ3339 exhibited progressive decreases in stress resistance and gene expression. These findings suggested that this classic gastrointestinal *S*. Typhimurium strain could sense and respond to changes in physiological fluid shear at levels naturally encountered in different host niches, which in turn might influence microbial responses during the infection process.

Unlike classic *S*. Typhimurium, which causes gastrointestinal disease, ST313 strain D23580 causes systemic infections^5,53^ and is thus exposed to higher fluid shear stress in the bloodstream.^37,38^ In previous work, D23580 cultured in the “standard RWV configuration” (*i.e.*, in the two orientations without the addition of beads) exhibited enhanced virulence potential and stress resistance in response to higher fluid shear conditions.^57^ Accordingly, it is critical to gain a mechanistic understanding of how physiologically relevant fluid shear forces associated with different host niche ecosystems during the infection process impact the pathogenesis of iNTS pathovars.

Herein, we investigated the influence of a broad range of incrementally increased fluid shear levels relevant to those encountered during the transition from the intestine to the bloodstream on diverse pathogenesis-related phenotypes, host-pathogen interactions, and global gene expression in D23580. A wide range of fluid shear levels were generated in the RWV by the addition of a series of beads with different sizes and densities. Mathematical modeling and computational simulations were used to quantify fluid shear levels in these hydrodynamic biosystems. Incremental increases in fluid shear increased the resistance of D23580 to acid and oxidative stresses, colonization of host cells, and survival within macrophages. In addition, incremental increases in fluid shear levels altered transcriptomic profiles in D23580, including upregulation of pathogenesis-related genes, including those in *Salmonella* Pathogenicity Island (SPI)-1 and SPI-4, which supported the observed phenotypic alterations. Collectively, the results of this study revealed a progressive relationship between physiological fluid shear levels, gene expression, and related pathogenic phenotypes of D23580. Our findings demonstrate how mimicking the mechanical force microenvironment to study host-pathogen systems biology can reveal emergent properties not possible using conventional approaches to advance our mechanistic understanding of infectious disease.

## Materials and methods

### Bacterial strains and growth conditions

ST313 strains were kindly provided by Dr Robert Kingsley (Wellcome Trust Sanger Institute, Wellcome Trust Genome Campus, Hinxton, Cambridge, UK) and Dr Robert Heyderman (Malawi-Liverpool-Wellcome Trust Clinical Research Programme). *S*. Typhimurium ST313 strain D23580 was cultured in Lennox broth (LB) at 37°C for all experiments. An overnight culture of D23580 grown for 15–16 h with aeration (180 rpm) was diluted 1:200 into fresh LB and loaded into the RWV bioreactors with or without beads as described below. The RWV was completely filled with culture medium, and air bubbles were removed to avoid additional fluid shear. The RWVs were incubated at 25 rotations per minute/rpm for 24 h with aeration to stationary phase (Supplementary Figure S1). Fluid velocities and shear stresses were calculated as described below.

### Generation of a range of incremental levels of fluid shear

**Figure 1.**
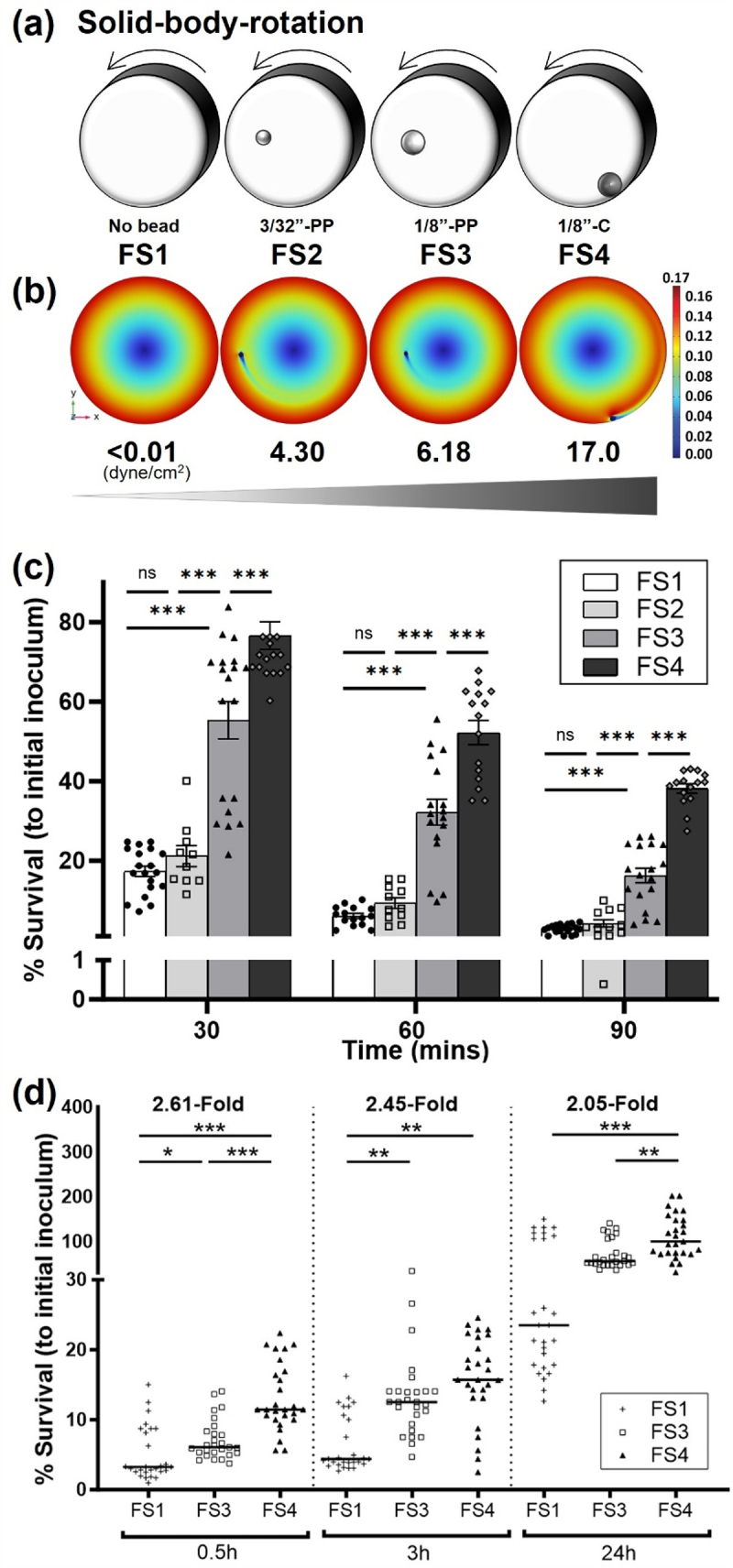
A series of incrementally increasing physiological fluid shear conditions progressively alters stress responses of D23580.

The RWV bioreactor (Synthecon) was used to generate different levels of fluid shear. Sterilized beads (3/32” diameter and/or 1/8” diameter) were included in the RWV during bacterial culture in order to increase fluid shear as previously described.^51^ The bioreactors used in this study were disk shapes (internal dimensions, 99.06 mm in diameter and 6.35 mm in thickness). Consequently, a single bead tends to fill most of the thickness dimension. Beads of differing sizes and densities were used to generate a range of incrementally increasing fluid shear levels in the RWV: 3/32” diameter polypropylene (**3/32” PP**); 1/8” diameter polypropylene (**1/8” PP**); and 1/8” diameter ceramic (**1/8” C**) (Baltec, Inc., Los Angeles, CA.). The fluid shear levels in the RWV with and without bead addition are shown in Figures 1 and 5. The steady-state Navier-Stokes equations were used to quantitate fluid shear levels in the RWV.^51,58^ The 1/8” ceramic bead, which is denser than the 1/8” polypropylene bead, was included to further increase the fluid shear. To generate an even higher fluid shear condition, two beads were added simultaneously to the RWV (1/8”-PP and 1/8”-C). For ease of reference, the five different incrementally increasing fluid shear conditions ranging from low-to-high as a result of single or multiple bead addition(s) in the RWV are designated as follows: Fluid shear 1 (**FS1; no bead**); Fluid shear 2 (**FS2; single 3/32” PP bead**); Fluid shear 3 (**FS3; single 1/8” PP bead**); Fluid shear 4 (**FS4; single 1/8” C bead**), and **FS3+FS4** (a combination of the **1/8” PP bead and** the **1/8” C bead**).

(a) Diagram of the RWV bioreactor showing spherical beads of different sizes and densities to induce incremental increases in fluid shear stress, from FS1 (Fluid Shear 1, < 0.001 dynes/cm^2^) to FS4 (Fluid Shear 4, 16.97 dynes/cm^2^). PP indicates polypropylene bead; C indicates ceramic bead. (b) Mathematical modeling of the fluid velocity distributions for calculating fluid shear levels experienced by bacteria exposed to FS1 to FS4 in the RWV bioreactor. The velocity (shown in meters/second on the scale bar) increased radially from the center of the bioreactor except in the region near the spherical bead. The bead-induced disruption of the velocity field is responsible for the elevated shear stresses in the fluid. (c) Percent survival at pH 3.5 of D23580 following growth in the RWV under incrementally increased fluid shear. (d) D23580 survival profile in J774 macrophages at different kinetic timepoints following bacterial growth in the RWV under incrementally increased fluid shear. Error bars indicate SEM. ANOVA was conducted along with Tukey’s multiple comparisons test (*p* < .001). Mean values, standard error of the mean, and p-values are indicated (*, *p* < .05; **, *p* < .01; ***, *p* < .001).

### Pathogenesis-related stress assays

Bacteria were grown as described above to stationary phase, and 10 mL of each culture was harvested and immediately subjected to the selected stresses. For acid stress assays, sterile 1 M citrate buffer was added to lower the pH to 3.5 as previously described.^49^ Cells were incubated statically at room temperature during exposure to each stress, and the pH was confirmed with an electrode at the end of the acid stress assay. Samples were removed at time zero (before the addition of stress) and at various time points thereafter, diluted in phosphate buffered saline (PBS) for the acid stress assay and plated on LB agar to determine the CFU/mL. Percent survival was calculated as the ratio of the CFU/mL at each time point to the CFU/mL at time zero. Three biological replicates were performed independently, and each included three technical replicates.

### Cell lines and culture conditions

The human colonic adenocarcinoma cell line, HT-29, was obtained from the American Type Culture Collection (ATCC, #HTB-38) and cultured in GTSF-2 media (Hyclone) containing 10% heat-inactivated fetal bovine serum (FBS) and 2.5 mg/L insulin/transferrin/sodium selenite supplement at 37°C in 10% CO_2_, as previously described.^59^ HT-29 cells were initially grown as monolayers in 75cc flasks until reaching confluency. Cells were then seeded into 24-well plates at a concentration of about 2 × 10^5^ cells/mL and grown to 100% confluency for use in infection studies. Trypan blue dye exclusion was used to determine cell viability. The mouse macrophage cell line J774A.1 (ATCC, #TIB-67 TM) was cultured in high glucose-Dulbecco’s Modified Eagle’s Medium (DMEM) (Gibco, #11995) containing 4 mM L-glutamine, 4500 mg/L glucose, 1 mM sodium pyruvate, and 3700 mg/L sodium bicarbonate with 10% heat-inactivated FBS. These cells were grown as monolayers at 37°C with 5% CO_2_ without antibiotics. The number of live cells was enumerated by trypan blue exclusion prior to infection.

### In vitro infections

D23580 was verified to be gentamicin-sensitive prior to the infection studies. The number of live cells (HT-29 or J774.1) was enumerated by trypan blue exclusion prior to infection and cells were infected with D23580 at a multiplicity of infection (m.o.i.) of 10 for 30 min. The infected cells were washed three times with Hanks’ balanced salt solution (HBSS) and lysed with 0.1% sodium-deoxycholate or incubated further in appropriate media (DMEM for J774.1 or GTSF-2 for HT-29 cells) with 50 μg/mL gentamicin for 3 hrs post-infection (h.p.i.) at 37°C with 5% CO_2_ for J774.1 or 10% CO_2_ for HT-29 cells. Three biological replicates were performed independently, and each included three technical replicates that were averaged for intracellular invasion. At 3 h.p.i., cells were then washed three times with HBSS and either lysed or further incubated in fresh media with 10 μg/mL gentamicin at 24 h.p.i. for intracellular survival/replication). The infected cells were then washed and lysed as previously described.^59^ Lysates were serially diluted 10-fold with sterile PBS and each dilution plated onto LB agar and counted to determine CFU/mL. Percent survival relative to the initial inoculum was calculated as a ratio of the CFU/mL at each time point to the CFU/mL of the infection dose at time zero. For each set of experiments, three independent biological replicates were conducted. Each biological replicate consisted of two to three technical replicates, the results of which were averaged. The J774 cell experiments were carried out over three independent trials, yielding a total of 27 observations (*n* = 27). For the HT-29 infection studies, two independent trials were performed, each comprising three biological replicates and two to three technical replicates, resulting in a total of 12 to 18 observations (*n* = 12 or 18). Data presented represent the average of all three biological replicates. Matched time point controls were included for all studies using the noninvasive *E. coli* strain HB101.

### RNA-Seq analysis

RNA-Seq was performed on an Illumina HiSeq-2000 sequencer to profile the entire D23580 transcriptome. Three RNA sample types were processed: no bead (FS1), 1/8” PP bead (FS3), and 1/8” C bead (FS4). For all three RWV conditions (FS1, FS3 and FS4), the RNA samples were prepared from two sets of biological replicates of D23580 cultures grown in LB medium to stationary phase for 24 hours at 37°C. Bacterial cells were fixed with RNA*later* (Qiagen). Total RNA was purified using the miRNeasy kit (Qiagen) and quantified using a Nanodrop spectrophotometer, and the integrity verified by formaldehyde-agarose gel electrophoresis. Sample cDNA libraries were generated utilizing the Encore complete prokaryotic RNA-Seq library systems. The barcodes used for RNA-Seq are listed in Supplementary Table S1. The synthesized cDNAs (generated with Nugen’s Ovation SPIA chemistry) were fragmented, ligated with adaptors using Kapa Biosystems NGS library preparation kit, and PCR-amplified using Kapa Biosystems HiFi polymerase to generate sequencing libraries. Six-plexed 2 × 100bp paired-end sequencing yielded a total of 202 million pairs of reads, and the reads per sample ranged from 28 to 48 million (Supplementary Table S2a). sA series of quality control metrics were generated on the STAR outputs. Stringtie-1.3.4c was used to report read counts, FPKM values (Fragments Per Kilobase of transcript per Million mapped reads) and TPM (Transcripts Per Million). RNA-Seq reads for each sample were quality checked using FastQC v0.10.1, aligned to the *S*. Typhimurium D23580 chromosome FN424405 and plasmid pST-BT FN432031, counted per gene, and quantified as FPKM values and TPM by TopHat and Cufflinks software.^60^ When data from duplicated samples for each group were merged, we detected an average of ~ 2,500 genes (out of ~ 4,500 protein coding genes in the genome) with FPKM > 1 with average coverage of ~ 50× (Supplementary Table S2b). Differential expression (DE) analysis was performed with EdgeR package from Bioconductor v3.2 in R 3.2.3. EdgeR applied an overdispersed Poisson model to account for variance among biological replicates. Empirical bayes tagwise dispersions are also estimated to moderate the overdispersion across transcripts. Then a negative binomial generalized log-linear model was fit to the read counts for each gene for all comparison pairs. For each pairwise comparison, genes with false discovery rate (FDR) <0.05 were considered significant and log2-fold changes of expression between conditions (logFC) were reported. False discovery rate (FDR) was also calculated following Benjamini & Hochberg (1995) procedure. Pathway enrichment was performed by DAVID 6.8 Functional Annotation Tool.^61^ Differentially regulated transcripts were analyzed for enrichment in GO terms and KEGG pathways using a threshold count of 2, an EASE score of 0.05 and Benjamini-Hochberg correction (<0.05).

### Statistical evaluation for stress assays and infection studies

All stress assays and infection assays were repeated at least three times. Each experiment included three technical replicates by plating samples onto LB agar in triplicate. ANOVA (α < 0.05) was conducted along with Tukey’s multiple comparisons test using Prism 9. Mean values, standard error of the mean (SEM), and p-values are indicated (*, *p* < .05; **, *p* < .01; ***, *p* < .001).

### Mathematical modeling of fluid velocities and shear stresses

Following the methodology developed by Nauman et al.. (2007), the measured equilibrium position of each bead in the RWV was used to construct the models of the bioreactor using a commercial software package, Comsol 6.1 (Comsol, Burlington, MA). Briefly, sectioning the RWV at the midpoint of its thickness between the front and back faces and denoting that face as a plane of symmetry, made it possible to decrease the solution time without sacrificing accuracy. Boundary conditions were then applied to the outside edges and back face of the bioreactor. Each was given the same angular velocity (25 rpm). The equilibrium faces of each bead were denoted as no slip conditions. Because the bacteria are small compared to the beads, it was assumed that they would not substantially disrupt the flow field. For this model, the fluid was assumed to be Newtonian with a density of 1,000 kg/m^3^, and a viscosity of 0.001 kg/(m*s). The velocity increased radially except for the region near the spherical bead. The equilibrium positions of the beads used in FS2 and FS3 were reported previously.^51^ For FS4 and FS3+FS4, the steady state position of the 1/8” diameter polymer bead was 35.5 mm from the center and 4.5 degrees below the horizontal. The ceramic bead sat at the edge of the bioreactor at an angle of 16 degrees from the vertical. Compared to the study by Nauman et al.,^51^ the resolution of these models was 80 times greater.

## Results

### Incrementally increasing fluid shear induces progressive increases in acid stress resistance and macrophage survival of D23580

To investigate the effect of different fluid shear levels that are physiologically relevant to those encountered by D23580 during infection, we first generated a series of incrementally increasing fluid shear conditions in the RWV, which we have designated as fluid shear conditions 1 to 4 (FS1 to FS4) (Figure 1(a)). Briefly, we applied our previously developed and validated quantitative biosystem, which incorporates small beads of different sizes and/or densities in the RWV bioreactor during bacterial culture in order to progressively increase fluid shear levels.^51^ This approach also facilitates mathematical modeling of the physiological fluid shear stress on bacterial cells in the RWV.^51^ In our current study, we further advanced this system to add higher fluid shear levels in order to broaden the fluid shear spectrum evaluated. The results from our modeling confirmed that maximum fluid shear stress was observed at the surface of the bead where the greatest velocity gradients were calculated (Figure 1(b)). The simulation results reported herein were performed at a considerably higher resolution than the previous studies^51^ with associated relatively minor changes in the peak fluid shear stresses. The maximum fluid shear stresses in FS1 to FS4 were <0.001 dynes/cm^2^ (no bead), 4.303 dynes/cm^2^ (3/32” polypropylene/PP bead), 6.178 dynes/cm^2^ (1/8” PP bead), and 16.97 dynes/cm^2^ (1/8” ceramic/C bead), respectively (Figure 1(b)).

We cultured D23580 using this biosystem and examined whether incremental changes in fluid shear differentially regulated the acid stress resistance of this pathogen (α < 0.05). Bacteria were grown in RWV bioreactors under the series of different FS conditions, harvested, and immediately subjected to pH 3.5, reflecting the upper limit of stomach acidity.^62–66^ Overall, D23580 displayed a progressive relationship (*p* < .001) between incrementally increasing levels of fluid shear and the acid resistance phenotype (Figure 1c). It should be noted that the FS2 condition did not show significant differences between FS1 for any timepoints, thus it was excluded from the study.

We next examined the internalization and intracellular macrophage survival profiles of D23580 cultured under these same fluid shear conditions (α<0.05). In general, the results showed that incremental increases in fluid shear progressively increased the colonization at 30 min (FS1<FS3<FS4), 3 hrs (FS1<FS3, FS1<FS4) and 24 hrs (FS1<FS4, FS3<FS4) post-infection of J774 macrophages (*p* < .001), which included a ≥ 2-fold difference between the FS1 and FS4 conditions at each time point (Figure 1(d)). To gain mechanistic understanding of the relationship between incremental increases in fluid shear and alterations of acid resistance and macrophage survival, we performed transcriptomic profiling of D23580 grown under FS1, FS3, and FS4 conditions.

### Fluid shear-induced alterations in the global gene expression profile of D23580 are associated with pathogenesis

**Figure 2.**
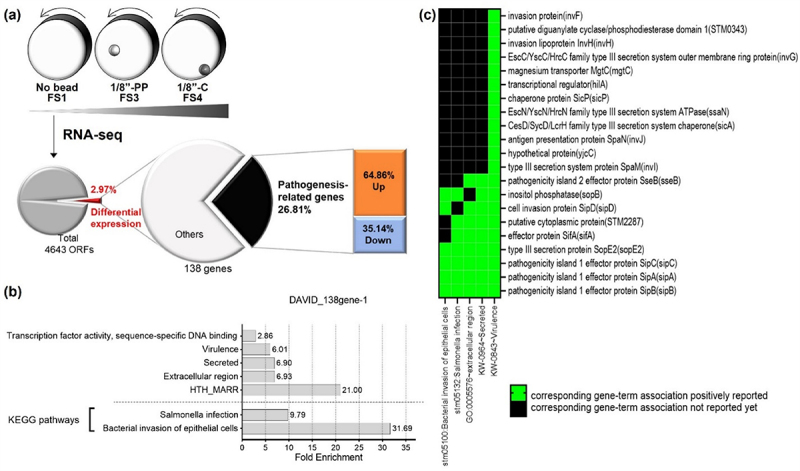
Comparative transcriptomic analysis of D23580 grown in different levels of fluid shear in the RWV.

To explore the impact of a range of physiological fluid shear levels on D23580 gene expression, we profiled changes in global expression of transcripts using RNA-Seq. Total RNA was purified from D23580 grown in the RWV under FS1, FS3, and FS4 conditions, sequenced, and evaluated for differential gene expression. A total of 4,643 open reading frames (ORFs) were found to be expressed across all conditions with 138 genes (2.97%) differentially expressed in both of the high fluid shear conditions (FS3 and FS4) relative to FS1 (*p* < .05) (Figure 2a and Supplementary Data). Of these 138 genes, ~27% were pathogenesis-related, including those associated with *Salmonella* Pathogenicity Islands (SPI-1, −4, and −5). Diverse functional families encoded by these genes were associated with invasion, adherence, iron acquisition, drug resistance, virulence, flagella, and lipopolysaccharide (LPS) (Figure 2a and Supplementary Data). Of the 37 pathogenesis-related genes that were differentially expressed, 64.86% (24 genes) were up-regulated while 35.14% (13 genes) were down-regulated (Figure 2(a)).

(a) Experimental diagram and pie chart showing the proportion of differentially expressed genes responding to different fluid shear (FS) levels. (b) Gene ontology analysis for the 138 differentially expressed genes in both FS3 and FS4, by using DAVID 6.8. (c) Functional annotation clustering analysis for the 138 genes whose expression changed in response to different levels of fluid shear stresses. Cut off was p-value < .05 between different FS conditions.

Gene ontology (GO) analysis of the 138 differentially regulated transcripts revealed significant enrichment (false discovery rate/FDR <0.05) of transcripts associated with sequence-specific DNA binding transcription factor activity, virulence, secretion, extracellular region, and helix-turn-helix Multiple Antibiotic Resistance R domain (HTH-MarR) (Figure 2b and Supplementary Table S3). Interestingly, KEGG pathway analysis revealed significant enrichment in pathways associated with bacterial invasion of epithelial cells and *Salmonella* infection (Figure 2(b)). The most enriched gene groups by functional annotation clustering analysis were *invFGHIJ*, *hilA*, *sicPA*, *sopBE2*, *sipABCD*, *sifA*, and *mgtC*, which are associated with the categories of virulence, bacterial invasion of epithelial cells, secretion, *Salmonella* infection and extracellular region (Figure 2c and Supplementary Fig. S2). Based on these results, we decided to take a closer look into how fluid shear modulated expression patterns of genes specifically associated with *Salmonella* virulence or pathogenesis.

**Figure 3.**
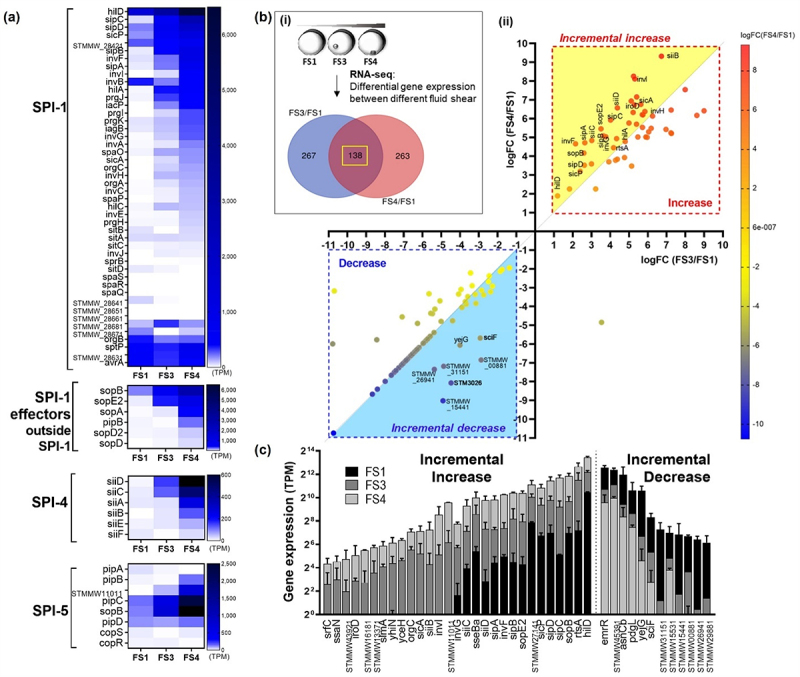
Comparison of differentially expressed genes between different fluid shear levels.

Gene expression levels of D23580 were calculated as TPM (transcripts per million) values and differential expression analysis was performed.^23,67,68^ We analyzed the genes in various SPIs that contain multi-gene loci critical for *Salmonella* infection and virulence.^54,69^ Our analysis showed that although some genes such as *sitD* of SPI-1 and *pipA* of SPI-5 exhibited downregulation with increasing fluid shear, there is a general trend of incrementally increased gene-expression patterns of SPI-1 (including secreted effectors), SPI-4, SPI-5 (Figure 3(a)). We therefore proceeded to analyze all differentially regulated genes to identify those demonstrating an incremental increase in gene expression correlating with incremental changes in fluid shear and *Salmonella* gene expression. We plotted the 138 common genes that were differentially expressed under the higher fluid shear conditions, comparing the log-fold changes between FS1 and FS3 (x-axis) to the log fold-changes between FS1 and FS4 (y-axis) (Figure 3(b)). Genes in quadrant 1 (red dotted box) were up-regulated in the higher fluid shear condition (FS3 or FS4 relative to FS1), while genes in quadrant 3 (blue dotted box) were down-regulated. Genes that were incrementally increased or decreased are highlighted in yellow or blue triangles, respectively (Figure 3(b)). Interestingly, we observed many pathogenesis-related genes were upregulated in response to incrementally increased fluid shear conditions, including *hilAD, invFGI, sipABCD, sopBE2, siiBCD*, *sicA*, and *rtsA*, showing incremental increases in expression (FS1<FS3<FS4) (Figure 3(b,c)).

(a) Heatmap of D23580 transcriptomes. Many genes in SPI-1, -4, -5, and SPI-1 effectors were upregulated when iNTS D23580 was grown in higher fluid shear conditions. (b) i) Venn Diagram showing the number of genes differentially expressed in different fluid shear conditions. ii) Scatter graph showing the fold changes in gene expression. The 138 genes were plotted for the graph. Each dot indicates genes showing differential gene expression in between FS1 and FS3 as well as in between FS1 and FS4 (*p* < .05). The dot color indicates the intensity of the fold difference, and the color scale is shown at the right. The x-axis shows the log-fold changes in gene expression by FS3 (the higher FS condition) over FS1 (the lowest FS). The y-axis shows the log-fold changes in gene expression by FS4 (the higher FS condition) over FS1. Details are included in the text. Red- or blue-dotted boxes indicate increase or decrease in gene expression. The yellow or blue shade indicates incremental changes in the gene expression, respectively. (c) Genes expressed in incrementally increased or decreased levels. The level of expression of individual genes was calculated as Transcripts Per Kilobase Million (TPM). *p* < .05

### Increased colonization of human intestinal epithelial cells is associated with higher fluid shear levels

**Figure 4.**
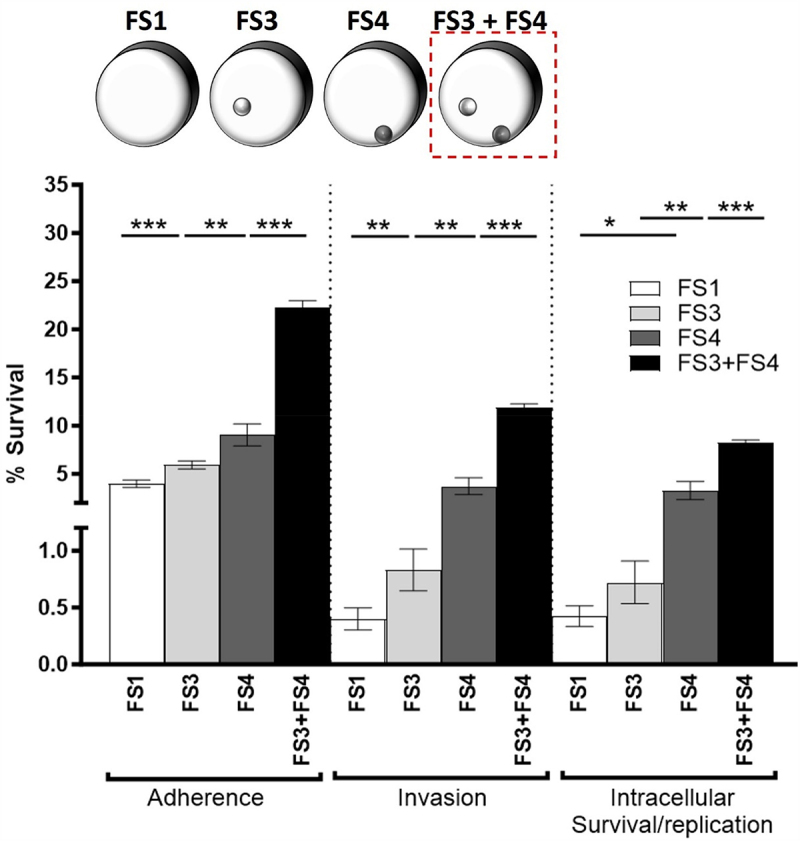
Addition of FS3+FS4 condition: *Salmonella* cellular responses to incrementally increased fluid shear levels from FS1 to FS3+FS4.

Our transcriptomic data showed stepwise increases in the expression of infection-related genes in response to incremental changes in fluid shear. To determine the correlation between these gene expression trends and the initial stages of enteric salmonellosis, we explored the effect of incrementally increasing fluid shear on infection kinetics using human intestinal epithelial cells. In response to increasing fluid shear conditions, progressive increases in D23580 adherence, invasion, and intracellular survival/replication were observed (*p* < .001) (Figure 4). Interestingly, the addition of multiple beads to the culture simultaneously (1/8” PP and 1/8” C beads) further enhanced the colonization levels at all kinetic timepoints (Figure 4).

Colonization of HT29 intestinal epithelial monolayers by D23580 grown in incrementally increasing fluid shear conditions. The diagram of a series of RWV systems from FS1 to FS3 + FS4 used in this test is shown at the top. The dotted square indicates the addition of FS3 + FS4. ANOVA was conducted along with Tukey’s multiple comparisons test (*p* < .001). Mean values, SEM, and p-values are indicated. (*, *p* < .05; **, *p* < .01; ***, *p* < .001).

### Mathematical and computational modeling of fluid shear

**Figure 5.**
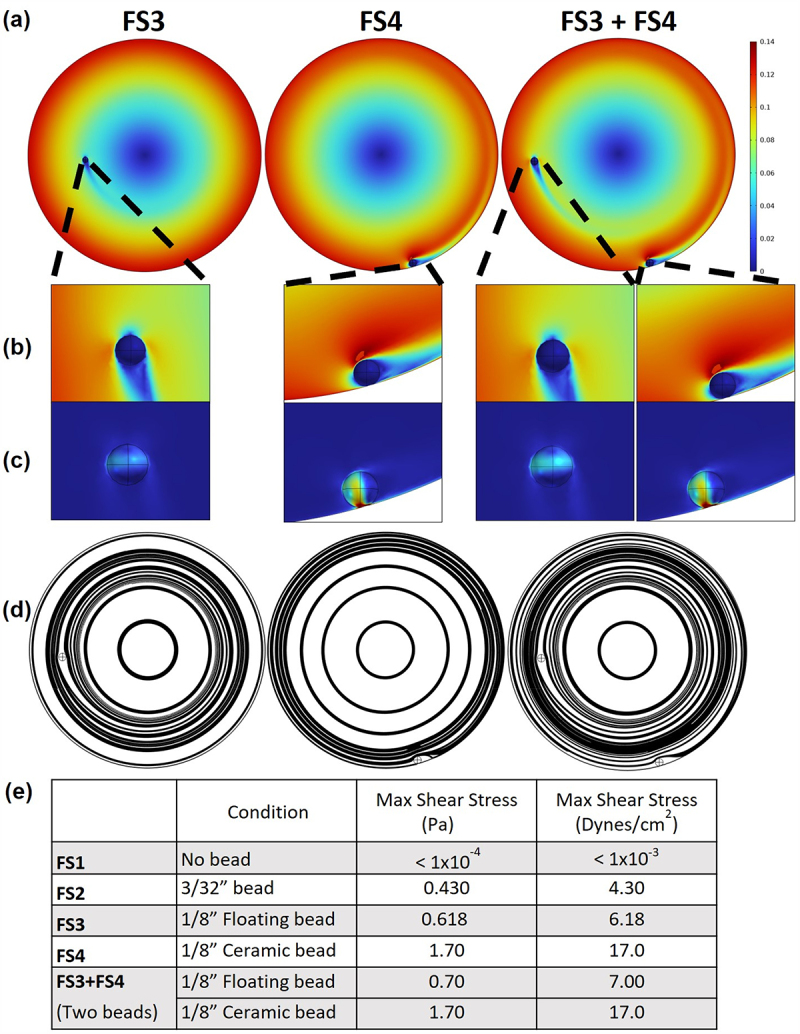
Quantification of the fluid velocity profile and fluid shear stress in the RWV for the FS3, FS4, and FS3 + FS4 conditions.

While we previously quantitated the fluid shear force environment in the RWV bioreactor for FS1-FS3 conditions,^51^ the fluid shear levels for FS4 and FS3 + FS4 used in this study had not been quantified. Accordingly, we quantified these fluid shear levels using the steady state Navier Stokes equations solved with Comsol 6.1 as previously described,^51^ but at 80 times the resolution of the simulations performed by Nauman et al..^51^ The velocity field data with a suspended bead in the FS3 condition (Figure 5a left) showed that the maximum fluid shear was observed at the surface of the suspended bead (Figure 5(b,c) left) and that the suspended bead disrupted the solid body rotation of the fluid (Figure 5d left). The fluid shear level for the FS3 condition was previously quantified to be 7.8 dynes/cm^251^ and the simulations performed here yielded a similar value of 6.178 dynes/cm^2^ (Figure 5(e)). In the FS4 condition (Figure 5 middle), which had a sedimented 1/8-inch ceramic bead rolling at the bottom of the RWV, (Figures 5a & 4), the current simulations estimated a value of 16.97 dynes/cm^2^ (Figure 5(e)). Fluid shear calculations for the FS3 + FS4 condition (Figure 5 right) with the combined suspended and rolling beads demonstrated that peak fluid shear stresses increased slightly for the suspended bead and were largely unchanged for the ceramic. Thus, the key finding from the FS3 + FS4 condition was that the volume of the bacterial culture that was exposed to the elevated levels of fluid shear stress was greater than the FS4 condition (Figure 5d).

(a) Velocity profile of the bioreactor demonstrated a maximum speed of 0.14 m/s at the outer edge of the bioreactor in each case. (b) Close up of the velocity profile near each of the beads. (c) The peak shear stress occurs in the fluid between the bead and the front and back surface of the bioreactor, but it should be noted that the peak shear stress on the ceramic bead was substantially higher than that on the suspended bead. (d) The streamlines illustrate the paths of theoretical, massless particles as the flow field transports them throughout the domain. The streamlines shown here demonstrate that the ceramic bead dramatically increases mixing within the bioreactor, and the combination of FS3 + FS4 exposes the greatest proportion of the bioreactor’s volume to elevated fluid shear stresses. (e) Maximum shear stress for each condition.

## Discussion

During their pathogenic existence, bacteria must sense, respond, and adapt to wide fluctuations in fluid shear forces in their natural environments, including in the infected host. However, most studies do not culture bacterial pathogens under physiological fluid shear conditions encountered in the host during the course of infection. This is a critical consideration, as fluid shear has been shown to profoundly alter phenotypic and molecular genetic responses of bacterial pathogens in unexpected ways.^35–39–41–45–49–57–70–75^ Indeed, culture of bacterial pathogens under physiological fluid shear forces has led to the identification of bacterial genes and proteins involved in host-pathogen interactions and infectious disease processes that have not previously been identified when these same organisms are grown conventionally in shaking or static flasks.^35–39–41–45–49–72,73–76^ While mechanical forces have long been recognized as vital in regulating the structure and function of mammalian cells,^77–79^ only recently has the importance of mechanobiology and how physical forces shape microbial pathogenesis begun to be more widely appreciated by the microbiology community.

The model enteric pathogen, *S*. Typhimurium, is one of the best characterized pathogens used to study the mechanobiology of infectious disease, especially as it pertains to the impact of physiological fluid shear on virulence and pathogenic mechanisms.^35–39–41–49–51–57–72, 73–82^ The majority of this research has been focused on the classical gastrointestinal disease-causing strain, χ3339, which showed that low fluid shear forces relevant to those encountered in the intestinal tract of the infected host increased the virulence and altered stress responses and gene expression of this organism.^41,49,72,73,82^ Given that closely related bacterial pathogens can respond differently to their microenvironment, it is important to understand how physical force cues can change pathogenic properties. However, the impact of physical forces on microbial pathogenesis and virulence remains poorly understood.

We previously performed an exploratory study to assess the impact of fluid shear on the virulence and basic stress response of *S*. Typhimurium ST313 pathovar, D23580, which causes serious and often fatal bloodstream infections, but does not routinely cause gastrointestinal disease.^5^ We showed that culture of D23580 under two different fluid shear levels (“standard RWV configuration” without beads) altered stress responses and virulence potential, leading to an earlier time-to-death in mice when this pathogen was pre-exposed to high fluid shear forces akin to the bloodstream.^57^ This was the opposite trend from that observed for χ3339, which typically causes gastrointestinal disease.^49^ However, the extent to which a broad range of physiological fluid shear forces can impact cellular and molecular genetic responses of D23580 remained unclear. In this study, we performed in-depth characterizations of a broad range of physiological fluid shear levels on the cellular and molecular pathogenesis properties of D23580.

Specifically, we modified the fluid shear capabilities of our validated quantitative biosystem^51^ to systematically examine the relationship between increased fluid shear forces and bacterial responses by introduction of quantitated incremental increases in fluid shear relevant to those encountered during the transition from the intestinal tract to the bloodstream. The fluid shear stresses used in our study (ranging from 0.001 to 16.96 dynes/cm^2^) are reflective of the physiological fluid shear stress levels encountered during this transition.^55,56,83–85^ Using this system, we found that phenotypic responses of D23580 directly correlated with incrementally increased fluid shear, including progressive alterations in acid stress resistance, macrophage survival, and colonization of human intestinal epithelial cells. In general agreement with these phenotypes, RNA-Seq analysis revealed a progressive upregulation of genes in SPI-1, −4, and −5 in response to incrementally increased fluid shear levels. SPI-1 is involved in host cell invasion and encodes a Type Three Secretion System (T3SS) that has several regulators, including HilD, HilC, HilA and RtsA.^86^ These transcription factors also regulate SPI-4 (encoding the SiiE adhesin known to enhance bacterial adherence to intestinal epithelium and induce inflammation^87–90^ and SPI-5 (encoding the SopB effector and other T3SS proteins).^86^ In addition, SPI-1 has been reported to regulate the differential polarization of macrophages toward the M2 phenotype which has low inflammatory and phagocytic activity and low anti-bacterial activity.^91^ Our findings suggest the possibility that in response to high fluid shear conditions relevant to those encountered in the bloodstream, D23580 may facilitate bacterial infections by upregulating the expression of genes in SPI-1, −4, and −5. Specifically, the mechanical force of high fluid shear in the bloodstream may facilitate production of the large non-fimbrial adhesin (encoded by SPI-4 *sii* genes), thereby enhancing *Salmonella* adherence to host cells. In addition, high fluid shear levels may also upregulate expression of SPI-1 protein regulators (such as those encoded by the *hil* and *rts* genes) and the SPI-1 T3SS apparatus (*inv* genes), thereby facilitating the delivery of SPI-5 and SPI-1 effector proteins (including those encoded by the *sip, sop*, and s*ic* genes), thus potentiating the infection. Collectively, these responses may contribute to the invasive disease caused by D23580. Interestingly, *rtsA* was differentially regulated in both D23580 and classic GI S. Typhimurium (χ3339) in response to different physiological fluid shear conditions.^41,51^ Studies are ongoing in our laboratory to characterize the role of RtsA in physiological fluid shear responses.

This is the first demonstration that incremental changes in physiological fluid shear levels alter pathogenesis-related stress responses, colonization of host cells, and global gene expression for any ST313 strain. Our results indicate that incremental increases in physiological fluid shear progressively alter mechanotransductive pathogenic phenotypes and global gene expression in *S*. Typhimurium D23580. Collectively, these findings advance our mechanistic understanding of how D23580 may dynamically modify its cellular and molecular responses to benefit fitness in high fluid shear conditions during systemic infection. This is a critical issue to address, as microbial sepsis is the third largest killer of humans globally. However, we have little understanding of the mechanisms underlying this important health problem, which is due in part, to a lack of knowledge regarding pathogen responses to the proper mechanical fluid shear environment. We are currently performing mutational analysis of targeted genes in D23580 to identify their specific roles in responding to the mechanical forces of fluid shear and their impact on disease outcomes.

## Supplementary Material

Supplemental Material

## Data Availability

The RNA-Seq datasets generated for this study can be found in the Gene Expression Omnibus (GEO) database (GSE241048) at https://www.ncbi.nlm.nih.gov/geo/query/acc.cgi?acc=GSE241048.
