# Supplementary material for: Incremental increases in physiological fluid shear progressively alter pathogenic phenotypes and gene expression in multidrug resistant Salmonella
Source: Gut Microbes. 2024 May 23;16(1):2357767. doi: 10.1080/19490976.2024.2357767 (PMC11135960; doi:10.1080/19490976.2024.2357767)
Supplement: Supplemental Material [file KGMI_A_2357767_SM5239.zip › Supplementary Figure 1.docx]

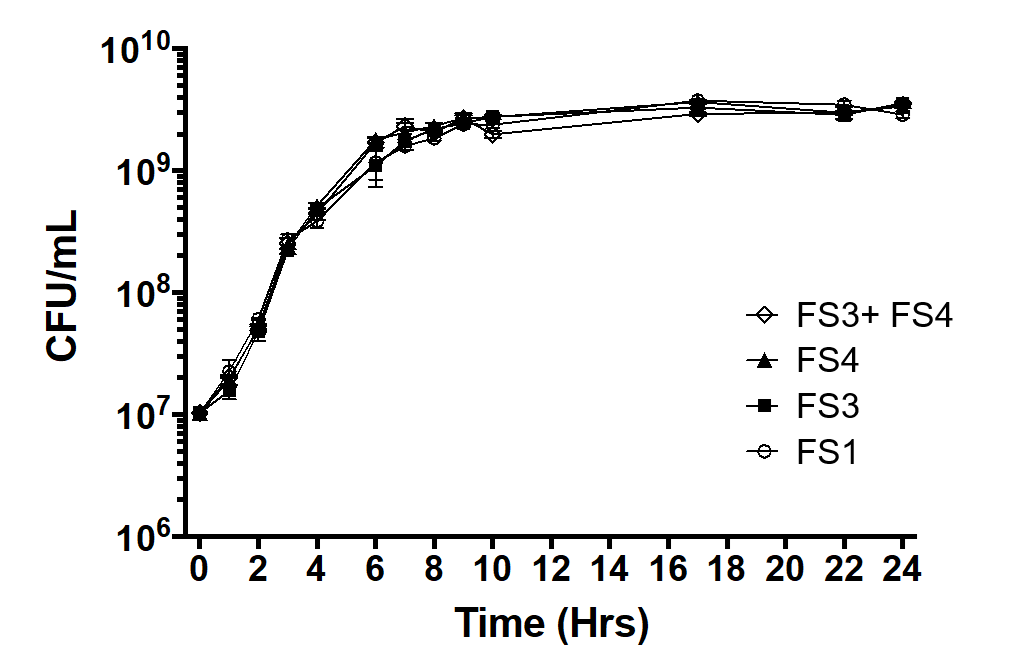


Supplementary Figure 1. Growth curves of D23580 grown in RWV bioreactors with or without beads. D23580 was pre-cultured in LB with aeration (180 rpm) for 15 hours at 37°C. These cultures were diluted at 1:200 in fresh LB and loaded into RWVs (~50 mL per bioreactor) with or without beads and subsequently incubated at 37°C and 25 rpm as described. Cultures were monitored for 24 hours by plating bacteria on LB agar for viable colony-forming units (CFU/mL). Error bars represent SD.
