# Supplementary material for: Incremental increases in physiological fluid shear progressively alter pathogenic phenotypes and gene expression in multidrug resistant Salmonella
Source: Gut Microbes. 2024 May 23;16(1):2357767. doi: 10.1080/19490976.2024.2357767 (PMC11135960; doi:10.1080/19490976.2024.2357767)
Supplement: Supplemental Material [file KGMI_A_2357767_SM5239.zip › Supplementary Table 1 (1).docx]

**Supplementary Table 1.** Barcodes used for RNA sequencing.

| **Label** | **Description** | **Barcodes** |
| --- | --- | --- |
| N1 | No bead sample 1 | CGATGT |
| N2 | No bead sample 2 | TGACCA |
| PP1 | 1/8” Polypropylene bead 1 | ACAGTG |
| PP2 | 1/8” Polypropylene bead 2 | GCCAAT |
| C1 | 1/8” ceramic bead 1 | CAGATC |
| C2 | 1/8” ceramic bead 2 | CTTGTA |
