# Supplementary material for: Incremental increases in physiological fluid shear progressively alter pathogenic phenotypes and gene expression in multidrug resistant Salmonella
Source: Gut Microbes. 2024 May 23;16(1):2357767. doi: 10.1080/19490976.2024.2357767 (PMC11135960; doi:10.1080/19490976.2024.2357767)
Supplement: Supplemental Material [file KGMI_A_2357767_SM5239.zip › Supplementary Table 2.docx]

Supplemental Table 2. RNA sequencing. N (no bead, FS1), PP (1/8” polypropylene bead, FS3), C (1/8” ceramic bead, FS4)

**(a) RNA-Seq read counts**

|  |  | Sample 1 | Sample 2 | Sample 3 | Sample 4 | Sample 5 | Sample 6 |
| --- | --- | --- | --- | --- | --- | --- | --- |
|  |  | **N1** | **N2** | **PP1** | **PP2** | **C1** | **C2** |
| Total paired Reads | | 28,733,271 | 29,829,775 | 48,462,255 | 27,930,881 | 35,214,995 | 32,034,443 |
| Mapped Reads | Count | 24,859,509 | 26,183,706 | 41,624,296 | 23,979,855 | 30,927,263 | 27,654,979 |
|  | % | **86.50%** | **87.80%** | **85.90%** | **85.90%** | **87.80%** | **86.30%** |
| Unmapped Reads | Count | 3,873,763 | 3,646,070 | 6,837,960 | 3,951,027 | 4,287,733 | 4,379,465 |
|  | % | **13.50%** | **12.20%** | **14.10%** | **14.10%** | **12.20%** | **13.70%** |

**(b) Gene counts and coverage**

|  |  | **N (FS1)** | **PP (FS3)** | **C (FS4)** |
| --- | --- | --- | --- | --- |
| Detected Genes | FPKM > 0 | 3005 | 2756 | 2620 |
|  | FPKM > 1 | 2731 | 2504 | 2300 |
| Ave. Coverage | | **51.2** | **67.6** | **38** |
