# Supplementary material for: Incremental increases in physiological fluid shear progressively alter pathogenic phenotypes and gene expression in multidrug resistant Salmonella
Source: Gut Microbes. 2024 May 23;16(1):2357767. doi: 10.1080/19490976.2024.2357767 (PMC11135960; doi:10.1080/19490976.2024.2357767)
Supplement: Supplemental Material [file KGMI_A_2357767_SM5239.zip › Supplementary Table 3.docx]

**Supplemental Table 3.** Gene ontology analysis and functional annotation clustering analysis for the 138 differentially expressed genes in both FS3 and FS4, by using DAVID 6.8.

| Category | Term | Gene Count | % | PValue | Genes | List Total | Pop Hits | Pop Total | **Fold Enrichment** | Bonferroni | Benjamini | FDR |
| --- | --- | --- | --- | --- | --- | --- | --- | --- | --- | --- | --- | --- |
| KEGG_PATHWAY | **stm05100: Bacterial invasion of epithelial cells** | 6 | 4.54545 | 3.21E-07 | sopB, sopE2, sipA, sipD, sipC, sipB | 37 | 9 | 1759 | **31.69** | 1.38E-05 | 1.38E-05 | 1.38E-05 |
| KEGG_PATHWAY | **stm05132: Salmonella infection** | 7 | 5.30303 | 4.24E-05 | sopB, sopE2, sseL, sipA, sipD, sipC, sipB | 37 | 34 | 1759 | **9.79** | 0.001822 | 9.12E-04 | 9.12E-04 |
| SMART | **SM00347: HTH_MARR** | 4 | 3.03030 | 4.74E-04 | emrR, hpaR, marR, STMMW_15461 | 26 | 6 | 819 | **21.00** | 0.009913 | 0.00996 | 0.00996 |
| GOTERM_CC_DIRECT | **GO:0005576~extracellular region** | 8 | 6.06061 | 1.00E-04 | sseL, sipA, sipD, sipC, sipB, sseBa, sifA | 72 | 41 | 2556 | **6.93** | 0.002602 | 0.002605 | 0.002605 |
| UP_KW_CELLULAR_COMPONENT | **KW-0964~Secreted** | 9 | 6.81818 | 2.44E-05 | sopB, sopE2, sseL, sipA, sipD, sipC, sipB, sseBa, sifA | 61 | 41 | 1917 | **6.90** | 2.93E-04 | 2.93E-04 | 2.93E-04 |
| UP_KW_BIOLOGICAL_PROCESS | **KW-0843~Virulence** | 21 | 15.90909 | 1.12E-11 | sopB, sopE2, sseL, sipA, sipD, sipC, sipB, sseBa, sifA, ssaN, sicP, invG, invF, invH, invJ, invI, yjcC, mgtC, STMMW_04131 | 55 | 110 | 1732 | **6.01** | 3.92E-10 | 3.92E-10 | 3.92E-10 |
| GOTERM_MF_DIRECT | **GO:0003700~transcription factor activity, sequence-specific DNA binding** | 15 | 11.36364 | 4.78E-04 | invF, hilD, emrR, sicA , orf408, marR, dgoR, rtsA, xapR, hpaR, slmA, STMMW_15461, STMMW_33561, STMMW_43921, STMMW_08861, STMMW_31351 | 83 | 194 | 3075 | **2.86** | 0.045279 | 0.046325 | 0.046325 |
