# Supplementary material for: Incremental increases in physiological fluid shear progressively alter pathogenic phenotypes and gene expression in multidrug resistant Salmonella
Source: Gut Microbes. 2024 May 23;16(1):2357767. doi: 10.1080/19490976.2024.2357767 (PMC11135960; doi:10.1080/19490976.2024.2357767)
Supplement: Supplemental Material [file KGMI_A_2357767_SM5239.zip › Supplementary_Figure_2.docx]

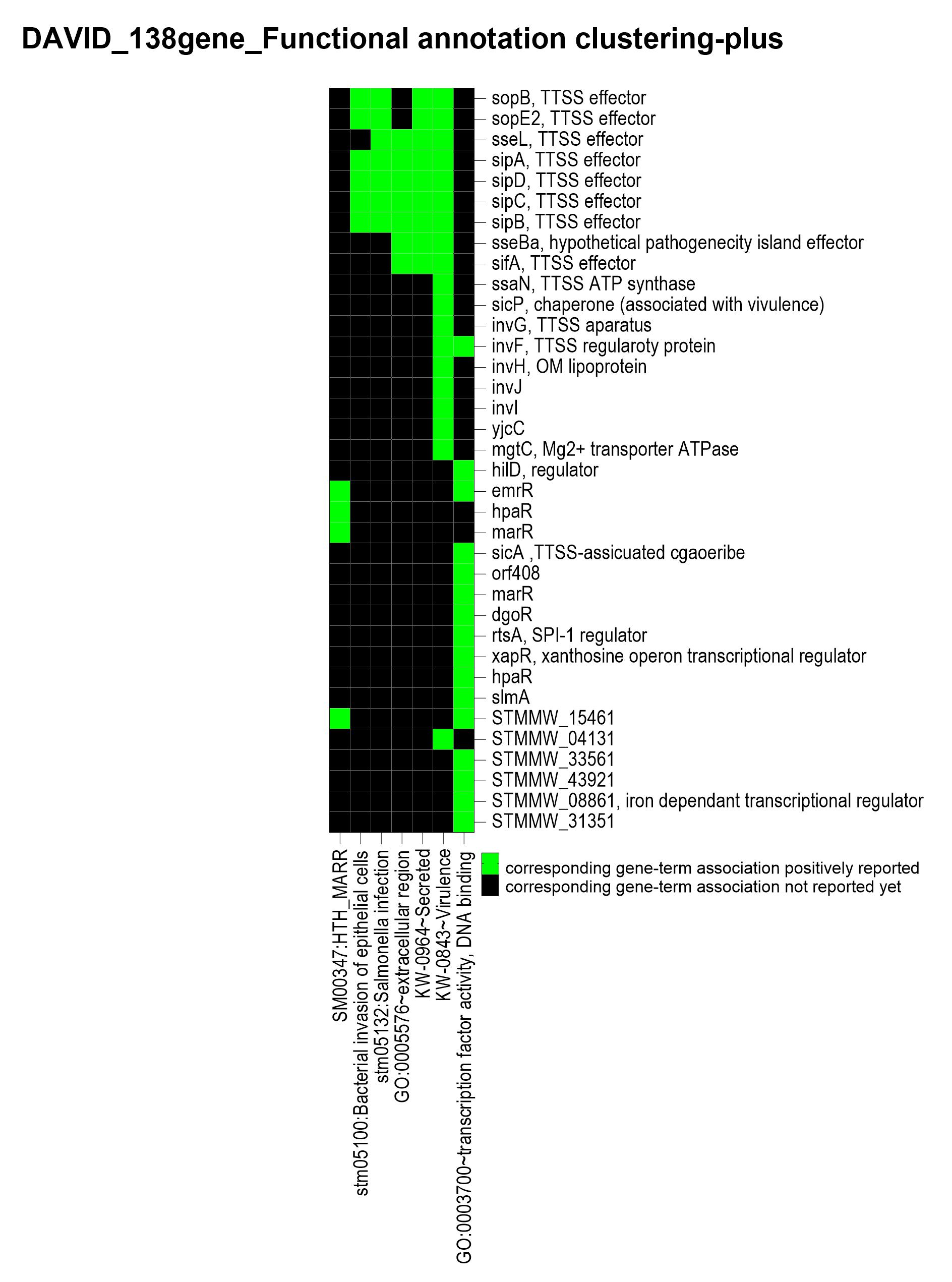


**Supplementary Figure 2.** Functional annotation clustering analysis for the 138 genes whose expression changed in response to different levels of fluid shear stresses. Cut off was p-value <0.05 between different FS conditions.
